# Prosystemin-derived signals: bridging leaf microbiome dynamics and defense activation

**DOI:** 10.1186/s40793-026-00885-9

**Published:** 2026-04-05

**Authors:** Valeria Castaldi, Wisnu Adi Wicaksono, Martina Chiara Criscuolo, Liberata Gualtieri, Emma Langella, Ilaria Di Lelio, Simona Maria Monti, Francesca De Filippis, Gabriele Berg, Rosa Rao

**Affiliations:** 1 Department of Agricultural Sciences, University of Naples “Federico II”, Portici, Italy; 2 Institute of Environmental Biotechnology, Graz University of Technology, Graz, Austria; 3 Institute for Sustainable Plant Protection - National Research Council (IPSP-CNR), Portici, Italy; 4 Institute of Biostructures and Bioimaging, National Research Council, Naples, Italy; 5 Present Address: Molecular, Cellular, and Developmental Biology Department, Yale University, New Haven, USA

**Keywords:** Signaling peptide, Plant microbiome, Phyllosphere, Shotgun metagenomics, Jasmonic acid, VOCs

## Abstract

**Background:**

Plant-derived peptides can act as resistance inducers and represent promising tools for sustainable crop protection. Despite growing interest and application, their broader effects on plant-associated microbiomes remain insufficiently characterized. Here, we investigated the impact of an immunomodulatory peptide derived from the tomato defense protein Prosystemin on the tomato phyllosphere microbiome and leaf volatilome.

**Results:**

The peptide was applied as a foliar spray at biweekly intervals from planting to two months post-germination to approximate common agricultural practices. Shotgun metagenomic sequencing combined with qPCR revealed abundant bacterial communities (up to 4.6 log_10_ bacterial 16S rRNA gene copies) dominated by Actino-, Alphaproteo- and Gammaproteobacteria across all samples. Peptide treatment was associated with a significant shift in community structure, characterized by reduced alpha diversity and increased microbial associations. Several genera, including *Acinetobacter*, *Sphingobium*, *Sphingomonas*, *Brevundimonas*, and *Massilia*, increased in relative abundance following treatment. Functional profiling indicated rearrangements in gene categories related to stress response and metabolic adaptation. Notably, volatilome analysis further revealed elevated monoterpene emissions in peptide treated plants, consistent with activation of defense-associated metabolism. Members of the Sphingomonadaceae family, particularly *Sphingobium yanoikuyae*, appear well suited to persist under peptide-associated conditions and may therefore contribute to the observed community restructuring, although causal mechanisms remain to be tested.

**Conclusion:**

Beyond its established role in protecting tomato against pests and necrotrophic fungi, the Prosystemin-derived peptide provides an opportunity to investigate peptide-triggered plant responses and their interactions with the plant microbiota.

**Supplementary Information:**

The online version contains supplementary material available at 10.1186/s40793-026-00885-9.

## Background

Agricultural practices aimed at protecting crops from abiotic and biotic stresses and promoting growth inevitably carry associated costs. Conventional chemical pesticides, while effective, can disrupt non-target organisms and alter ecological balance. Addressing these challenges requires integrated strategies that safeguard agricultural productivity and food security while minimizing environmental and health risks [1]. The intrinsic ability of plants to respond to stress offers a promising strategy to align crop protection approaches with natural defense mechanisms [2–4]. For instance, plant-derived peptide-based technologies are attracting increasing interest due to their multifunctional properties, ranging from immune priming to growth promotion [5, 6]. Some peptides directly target pests through antimicrobial, insecticidal, or nematicidal effects [6–8], while others, known as phytocytokines, function as signaling molecules that coordinate immunity or growth [9–12]. Tomato Prosystemin (ProSys) represents a valuable source of bioactive signaling peptides [13, 14]. Acting as a hub protein, ProSys contains functional motifs capable of coordinating gene expression under different environmental challenges, thus contributing to resistance against a wide range of pests [15, 16]. However, the plant holobiont perspective has reshaped the definition of plant resilience [17, 18], emphasizing that host genotype and associated microbial communities jointly influence plant performance [19–21]. The plant microbiota varies across compartments and developmental stages and is shaped by both vertical transmission and environmental inputs [22]. Members of the phytobiome contribute to plant health by suppressing phytopathogens [23, 24], enhancing nutrient uptake [25–27], modulating phytohormone pathways [28, 29], promoting germination and growth [30, 31], and degrading harmful compounds [32, 33]. This symbiotic functional interplay can be explained by plant‒microbe coevolution, with host genotype as a key determinant of community structure [34]. In this context, signaling peptide application may alter host physiology and favor microbial taxa adapted to the modified host environment [35–38]. Beyond transcriptional reprogramming, peptide-induced defenses can trigger broader metabolic changes, including the emission of volatile organic compounds (VOCs) which mediate ecological interactions across multiple scales [39].

Previous work revealed that ProSys-derived peptides mimic biotic stress, inducing a primed defensive state in tomato through the activation of jasmonic acid (JA)-related pathways [13, 14]. This response confers resistance to insects such as *Spodoptera littoralis* and necrotrophic fungi like *Botrytis cinerea* and *Alternaria alternata* [13, 14, 40]. Open-field trials in tomato further revealed increases in biomass, yield, and fruit quality following ProSys peptides application [41], supporting their agronomic potential. Based on these findings, we hypothesized that repeated application of ProSys-derived peptides may also be associated with shifts in leaf-associated microbial communities that contribute to plant resilience [20, 42, 43]. To test this, we performed shotgun metagenomic sequencing of tomato leaves after treatment with a specific ProSys-derived peptide, termed G [13], and compared microbial composition and functional potential with PBS-treated (CTRL) and untreated (UNT) plants. We focused on the leaf phylloplane as a primary interface of foliar agricultural treatments and a relatively underexplored niche [44]. Moreover, we profiled volatile emissions from G-treated plants to capture metabolic changes following peptide treatment and assess whether similar patterns emerged alongside the observed microbiome shifts.

## Results

### Prosystemin peptide alters bacterial community structure and diversity in the tomato phyllosphere

ProSys contains several repeated peptide motifs distributed along its sequence [13]. To determine whether the bioactivity of these motifs is sequence-specific, we identified three peptide stretches located outside the predicted functional motifs [13], hereafter referred to as negative control sequences (NCs). Their amino acid sequences and positions within ProSys are summarized in Additional file 1: Fig. S1A. Bioassays showed that none of the NCs triggered detectable defense responses in tomato plants.

In particular, NCs treatments neither induced the expression of JA-related defense genes (Additional file 1: Fig. S1B) nor affected larval performance in feeding assays with the polyphagous *Spodoptera littoralis* larvae (Additional file 1: Fig. S1C–D; Additional file 2: Supplementary Methods). These results indicate that ProSys bioactivity is sequence-specific and restricted to defined regions of the protein.

**Fig. 1:**
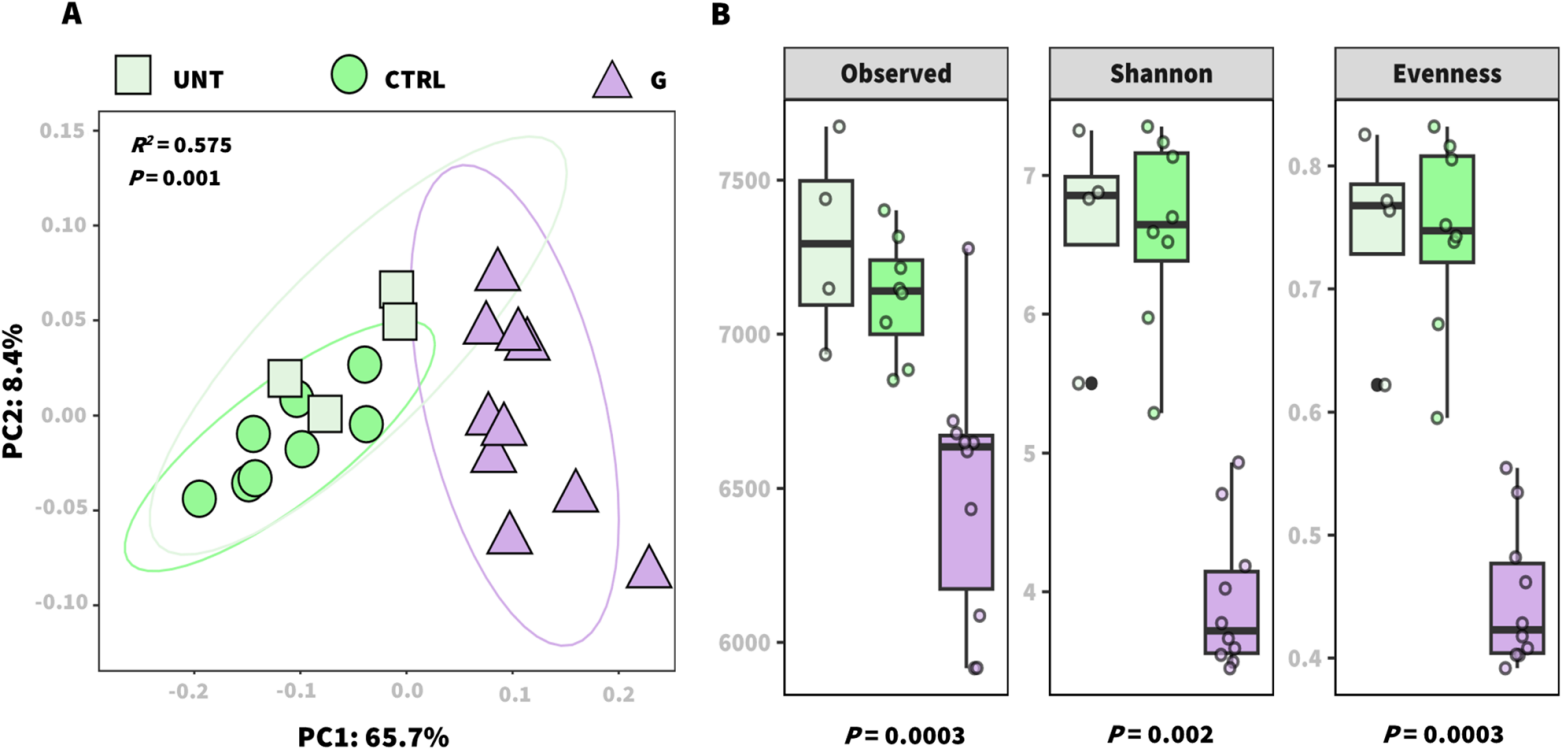
**Bacterial structure and diversity of the tomato leaf epiphytic community in untreated, control (PBS 0.1X), and G peptide-treated (100 fM) plants.** Bray-Curtis distance matrices of bacterial community structures between samples were visualized using a two-dimensional PCoA plot (**A**). Differences in community composition among groups were tested by PERMANOVA. Richness (observed species), Shannon, and Pielou's evenness indices (**B**) were calculated to explore the overall alpha diversity within the samples and statistical significance was evaluated using the Kruskal-Wallis test. UNT: untreated, CTRL: control-, G: peptide-treated plants.

We therefore investigated whether the application of the functional ProSys-derived G peptide could influence the structure of the tomato phyllosphere microbiome using shotgun metagenomics. In total, 768,712,768 high-quality reads were obtained. After host genome removal, 19,605,952 bacterial reads remained (Additional file 3: Table S1), representing 9,179 bacterial species across all samples. Compared with the CTRL and UNT treatments, peptide application significantly altered the overall bacterial community structure at the species level (Fig. 1A) (*R*^*2*^ = 0.575; *P* = 0.001). This shift was associated with a marked reduction in species richness (*P* = 0.002) and diversity (*P* < 0.001), together with changes in evenness (*P* < 0.001) according to the alpha diversity metrics (Fig. 1B). These results suggest that peptide application imposes selective pressure on the phyllosphere microbiome, favoring the proliferation of specific bacterial taxa over others.

### Alphaproteobacteria and Gammaproteobacteria dominate the peptide-treated leaves

**Fig. 2:**
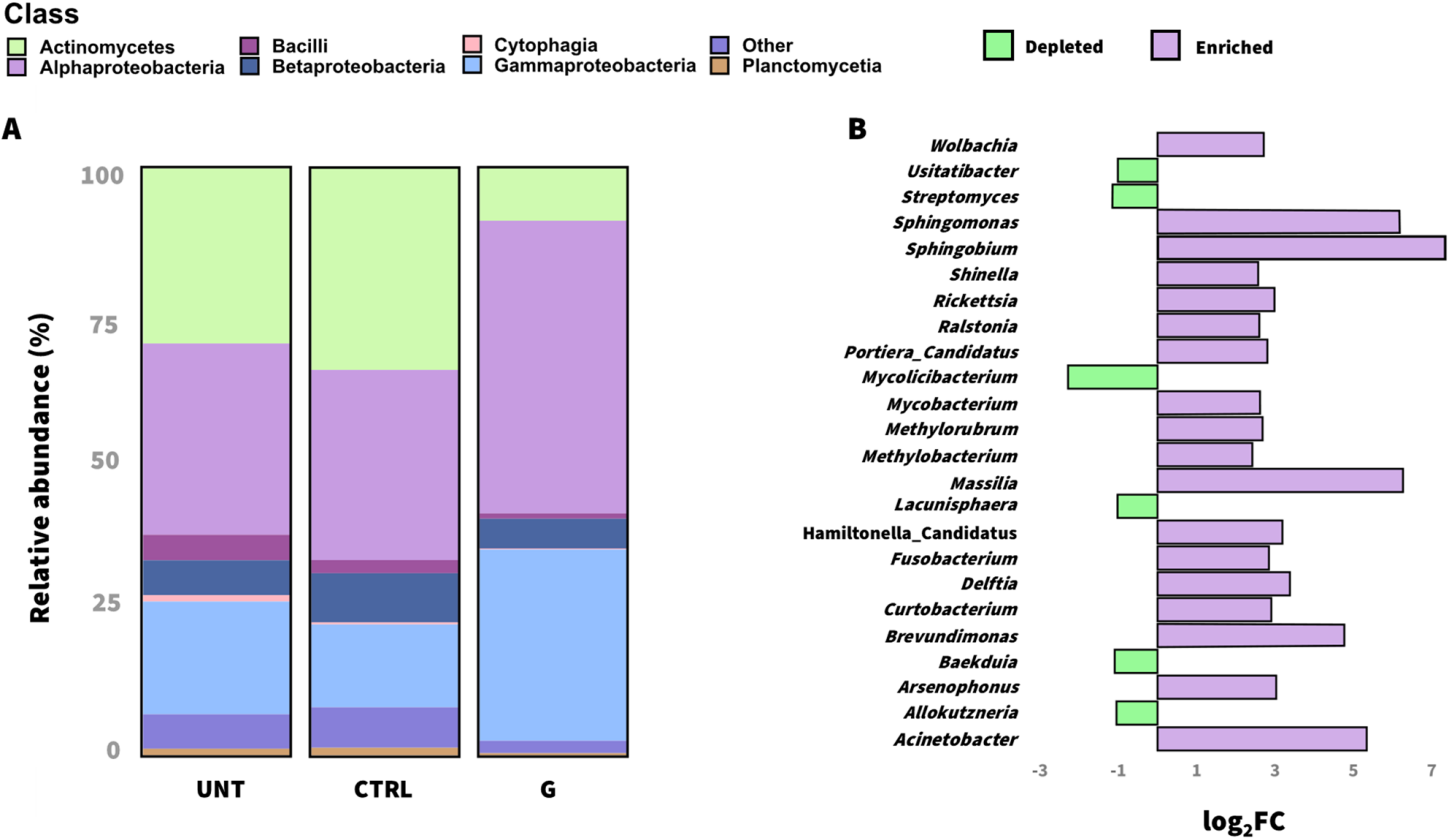
**Bacterial community composition of *S. lycopersicum* cv. San Marzano nano leaves subjected to different foliar spray treatments.** Mean relative abundance of bacteria at class level (**A**) in tomato plants under three different treatments: UNT (untreated), CTRL (0.1X PBS), and G peptide-treated (100 fM). Differential abundance analysis (**B**) performed at the bacterial genus level in the G-treated plants compared with the CTRL plants via the edgeR package. Positive log_2_-fold-change (log_2_FC) values (violet bars) indicate higher abundances of the respective bacterial genera in the G-treated samples, whereas negative values (green bars) represent higher abundances in the PBS-treated plants. Significantly enriched or depleted bacteria (*P*_*adjusted*_ < 0.05) with a relative abundance > 0.05% are shown.

Given the observed shifts in community composition, we analyzed changes in the relative and differential abundance of bacterial taxa. At the class level (Fig. 2A), peptide-treated leaves showed a strong increase in Alphaproteobacteria (48.3%) and Gammaproteobacteria (31.8%) compared with CTRL (32.7% and 15.9%, respectively) and UNT plants (29.7% and 16.9%, respectively). In contrast, Actinomycetes, which dominated both control groups (33%), were markedly reduced in the peptide-treated plants (10.1%). Because no significant differences were detected between CTRL and UNT plants in observed richness, Shannon diversity, or beta diversity (*P* = 0.08; *P* = 1; *P* = 0.1, respectively), subsequent analyses were focused on comparing G-treated and PBS-treated samples.

Differential abundance analysis using edgeR identified 1,612 bacterial species with positive log_2_-fold-change (log_2_FC) and 933 with negative log_2_FC in G-treated samples. Species were considered enriched or depleted when log_2_FC > 2 or < − 1 (*P* < 0.05) and retained when relative abundance exceeded 0.05% (Fig. 2B). Fold-changes values, relative abundances across individual samples, and treatment means are provided in the Additional file 3: Table S2.

**Fig. 3:**
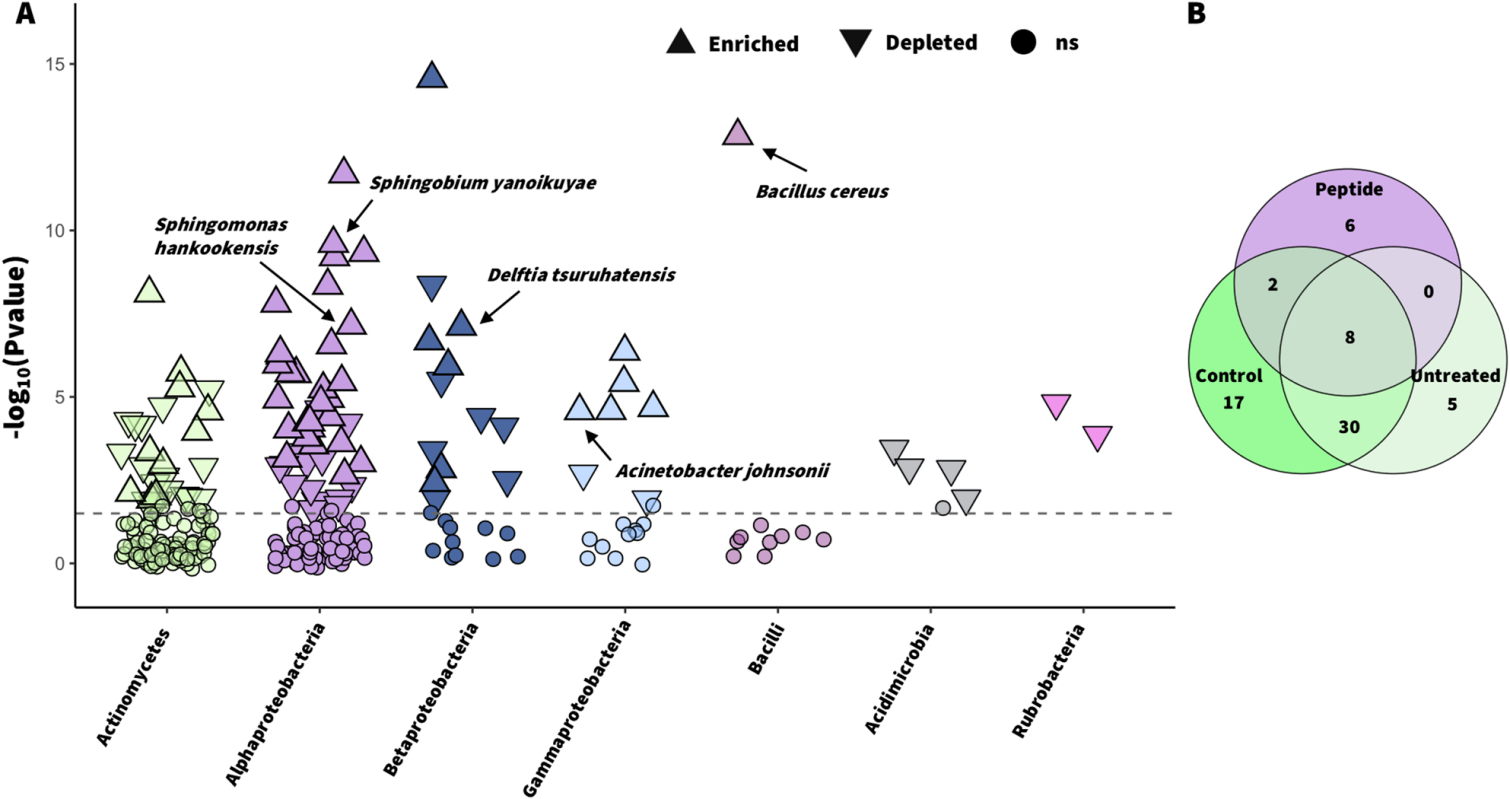
**Enriched bacterial species in the G treatment group compared with those in the CTRL group and a Venn diagram showing the core and unique number of species among the treatment groups.** Manhattan plot (**A**) showing enriched (upward-pointing filled triangles), depleted (downward-pointing filled empty triangles), and nonsignificant (ns, filled circles) bacterial species in the peptide-treated plants compared with the control (PBS). Species were filtered by *P* < 0.05 and a relative abundance (Ab) > 0.01%. Shared and unique core bacterial species among the three treatments (**B**) are shown with a Venn diagram. Core species were filtered by prevalence (75%) across samples and Ab > 0.1%.

Among Alphaproteobacteria, several genera were significantly enriched, including *Brevundimonas* (log_2_FC = 4.0, *P* < 0.001), *Sphingobium* (log_2_FC = 6.28, *P* < 0.001) and *Sphingomonas* (log_2_FC = 4.63, *P* < 0.001). Among Gammaproteobacteria and Betaproteobacteria, *Acinetobacter* (log_2_FC = 5.03, *P* < 0.001) and *Massilia* (log_2_FC = 6.27, *P* < 0.001) were the most enriched, respectively. At the species level, *Sphingobium yanoikuyae* (log_2_FC = 7.33), *Sphingomonas hankookensis* (log_2_FC = 6.13), and *Acinetobacter johnsonii* (log_2_FC = 4.35) showed significant increase in the peptide-treated plants (Fig. 3A) and were among the core bacterial species associated with this treatment (Fig. 3B). Interestingly, the relative abundance of several bacterial genera recognized as insect endosymbionts also increased following peptide treatment (Fig. 2A). Among them, *Rickettsia* (log_2_FC = 2.93, *P* < 0.001) and Hamiltonella_Candidatus (log_2_FC = 3.18, *P* < 0.001) were the most enriched.

In contrast, *Streptomyces*, one of the most abundant genera in CTRL plants (9.23%) was remarkably affected by peptide application (1.94%). Several *Streptomyces* species were also part of the core microbiome of both control conditions (Fig. 3B; Additional file 3: Table S3). Overall, these results indicate that G peptide treatment may favor taxa better adapted to the new phyllosphere conditions (e.g. Proteobacteria), thereby altering the equilibrium originally observed in both control conditions.

### qPCR reveals a higher bacterial load in G samples and strengthens the importance of few taxa

**Fig. 4:**
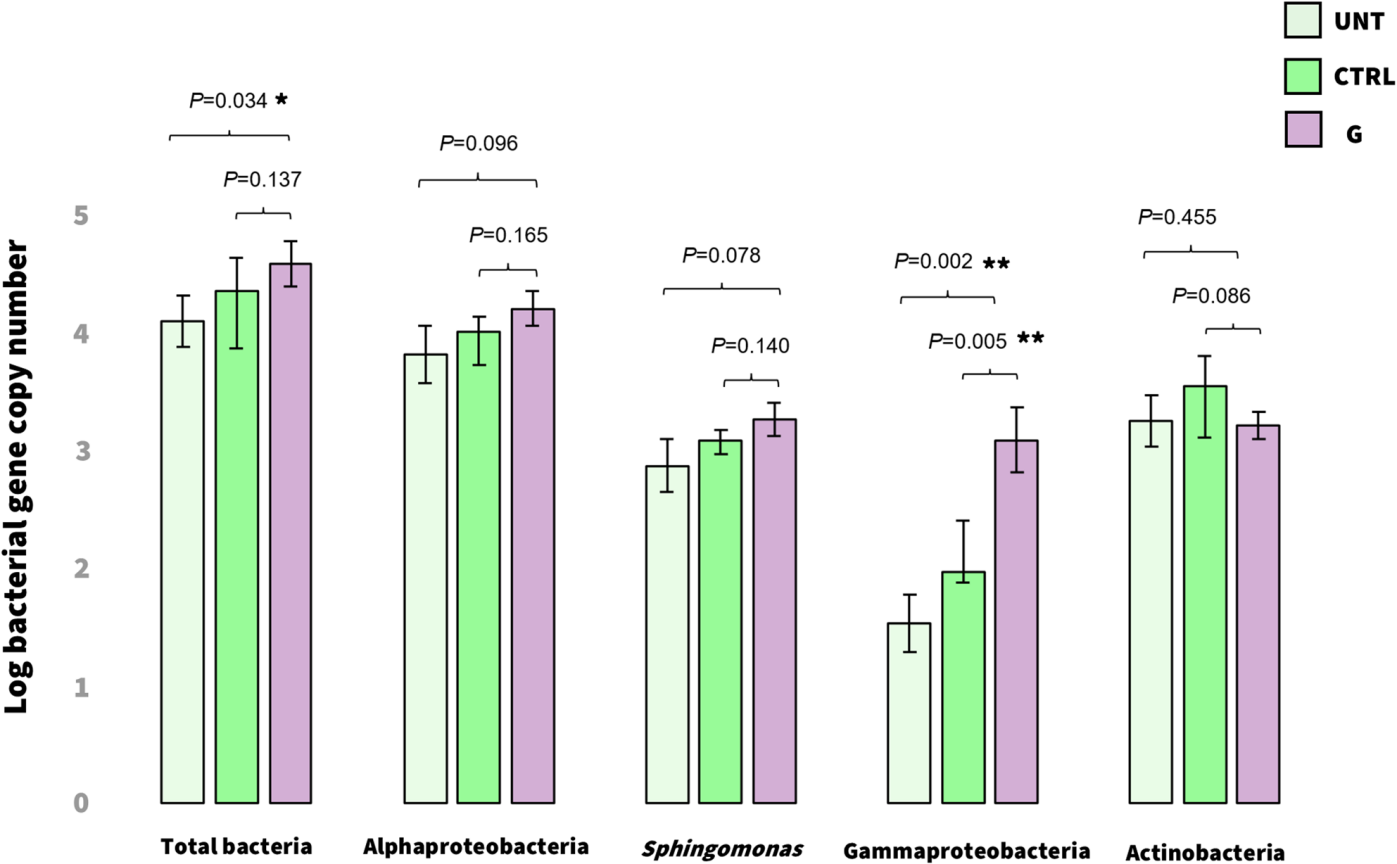
**Absolute abundances of total bacteria, Alphaproteobacteria, *Sphingomonas*, Gammaproteobacteria and Actinobacteria calculated with quantitative real-time PCR (qPCR) between untreated (UNT), PBS 0.1X-treated (CTRL), or peptide-treated (G) tomato leaves.** Statistical significance was assessed by Kruskal–Wallis with Dunn’s test for multiple comparisons (*P < 0.05, **P < 0.01). Error bars indicate the standard error.

To validate the metagenomic evidence of peptide-driven microbial enrichment, we quantified total bacterial load and the abundance of selected taxonomic groups by qPCR targeting 16S rRNA gene. Absolute bacterial abundance in UNT and CTRL leaves was 4.12 ± 0.4 and 4.37 ± 0.5 log_10_  16S rRNA gene copies, respectively (Fig. 4). Leaves treated with the G peptide showed a higher bacterial load (4.60 ± 0.4 log_10_ bacterial 16S rRNA gene copies) compared with UNT leaves. Although not statistically significant, similar trends were observed for Alphaproteobacteria and *Sphingomonas*, which displayed higher abundances in G-treated leaves (4.22 ± 0.3 log_10_ and 3.28 ± 0.3 log_10_ copies, respectively) relative to UNT (3.82 ± 0.5 log_10_ and 2.88 ± 0.4 log_10_ copies) and CTRL leaves (4.03 ± 0.2 log_10_ and 3.10 ± 0.2 log_10_ copies).

Notably, Gammaproteobacteria were significantly enriched in G-treated plants (3.10 ± 0.5 log_10_ copies) compared with UNT (1.53 ± 0.5 log_10_) and CTRL (1.97 ± 0.8 log_10_). In contrast, Actinobacteria were more abundant in CTRL treatment than in G-treated or UNT leaves. These trends showed consistency with the patterns observed in the shotgun metagenomic analyses.

### Peptide-induced restructuring of bacterial associations

**Fig. 5:**
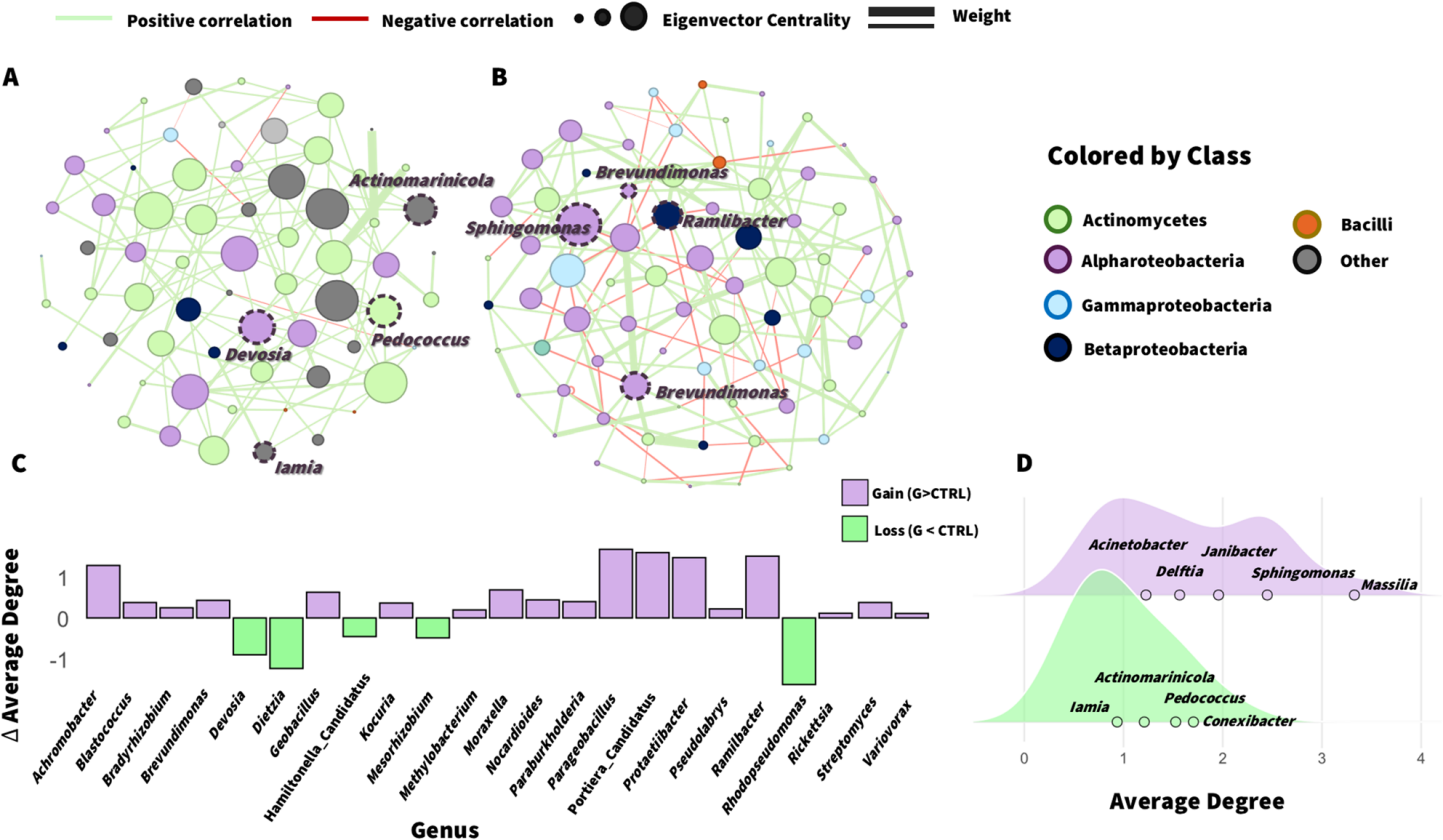
**Bacterial intra-kingdom co-occurrence networks in the control and ProSys-derived G peptide-treated tomato leaves.** Each node represents a bacterial taxon (colored by class), and edges indicate significant associations (green = positive; red = negative) in control (**A**) or G-treated (**B**) communities. Edge thickness and node size are proportional to association weight and eigenvector centrality, respectively. Hub nodes are highlighted with a purple dashed contour. Changes in average node degree between conditions (**C**), showing taxa that gained (purple) or lost (green) connectivity following peptide treatment. Distribution of average degree for genera exclusively detected in the control or peptide network (**D**). Density ridges indicate the overall connectivity pattern, with most representative genera labeled.

To explore how G peptide influences bacterial interactions on the phylloplane, we reconstructed two intra-kingdom co-occurrence networks using the SPRING algorithm. The control network (Fig. 5A) exhibited a fragmented and highly modular architecture, consisting of 21 independent components with high modularity (0.85) and a predominance of positive correlations (96.8%) (Additional file 3: Table S5). The proportion of nodes belonging to the largest connected component (LCC) was 0.19, indicating that only a small fraction of taxa was interconnected within the main network structure, while most formed smaller independent subnetworks. Within the LCC, hub identification revealed members of Actinobacteria, Acidimicrobiia, and Alphaproteobacteria, including *Pedococcus*, *Actinomarinicola*, *Iamia*, and *Devosia* (Fig. 5A). In contrast, the G peptide-associated network displayed a larger and more cohesive topology, with most taxa connected within a single dominant component (LCC = 0.93), comprising 78 nodes and 182 edges. Taxonomic profiling indicated enrichment of Alphaproteobacteria (46.8%) and reduction of Actinomycetes (29.9%) compared with CTRL (39.4%), suggesting Proteobacteria-driven associations in the new leaf environment. Although modularity (0.70) and clustering coefficient (0.34) were lower than in the CTRL, the G network exhibited a higher proportion of negative edges (22.3%) (Additional file 3: Table S5). Notably, *Brevundimonas* spp. and *Sphingomonas hankookensis* (Alphaproteobacteria), together with *Ramlibacter tataouinensis* (Betaproteobacteria), emerged as central hubs characterized by high eigenvector centrality and cross-module connections that integrated otherwise distant taxa (Fig. 5B). To compare connectivity patterns between conditions, we quantified the change in average node degree for shared taxa (Fig. 5C). Several genera, including *Achromobacter*, *Ramlibacter*, and *Brevundimonas*, gained connectivity following peptide treatment, while others such as *Devosia*, *Dietzia*, and *Rhodopseudomonas* showed reduced associations. This shift highlights a transition from Actinobacteria- to Proteobacteria-dominated network. In addition, analysis of exclusive genera (Fig. 5D) revealed that *Actinomarinicola*, *Iamia*, *Pedococcus*, and *Conexibacter* were uniquely detected in the control network, whereas *Massilia*, *Sphingomonas*, *Delftia*, *Acinetobacter*, and *Janibacter* were exclusive to the peptide-treated plants. The higher average degree of peptide-responsive taxa suggests that peptide exposure promotes the emergence of newly connected bacterial members.

The increased representation and connectivity of Proteobacteria, characterized by high metabolic flexibility and rapid responses to environmental changes, contrasts with the decline of Actinobacteria, which are more frequently associated with stable and oligotrophic niches [45, 46]. Consistently, the higher proportion of negative correlations observed in the peptide network may reflect stronger competitive interactions and niche differentiation among taxa adapting to the modified host environment. However, co-occurrence links should be interpreted as statistical associations rather than direct ecological interactions.

### Functional profiling indicates enhanced bacterial activity under changing environmental conditions

**Fig. 6:**
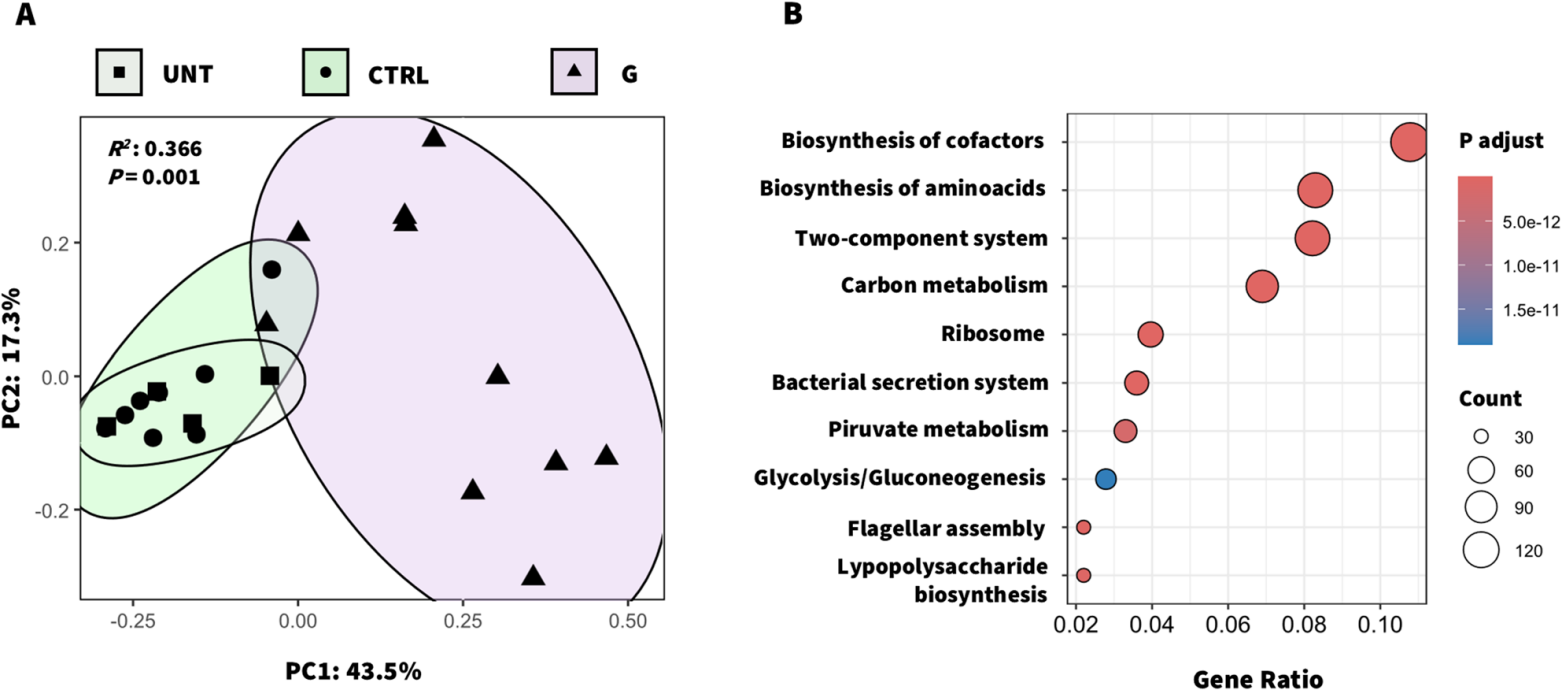
**Functional exploration of the leaf-associated bacterial community.** A Bray‒Curtis matrix (**A**) was used to visually explore the differences between the genes of the phyllosphere samples according to the different treatments. Top 10 KEGG pathways (**B**) based on the KEGG Orthology terms (KOs) associated with the enriched genes of the peptide-treated samples. The circle sizes represent the number of enriched-KOs grouped according to KEGG pathways (third level). The analysis was performed by the Microbiome Profiler package in R,

We next investigated whether peptide-driven taxonomic shifts were accompanied by functional changes by performing a functional profiling of bacterial genes and their pathways. High-quality filtered contigs were clustered together to identify potential protein-coding regions (ORFs). Among the 381,825 predicted ORFs, 189,644 were successfully annotated, and 56.7% were assigned to KEGG Orthology terms (KEGG-KO). Beta diversity analysis (Fig. 6A) confirmed that observed shifts in bacterial community composition were also associated with functional differences between treatments (*R*^*2*^ = 0.366, *P* = 0.001). According to KEGG-KO enrichment analysis (log_2_FC > 2, *P*_*ajdusted*_ < 0.05), the G-treated samples were enriched in genes associated with metabolic processes and environmental responses (Fig. 6B).

Specifically, genes involved in the synthesis of cofactors (147 KOs) and vitamins, including B12 (M00122, M00924, M00925), B6 (M00124) and B7 (M00123), were more abundant in G-treated plants. Genes involved in the biosynthesis of cofactors associated with energy metabolism and redox balance were also enriched, including pathways for tetrahydrofolate (M00841, M00842), coenzyme A (M00120), coenzyme Q (M00117), and molybdenum (M00880). Notably, higher detection of amino acid biosynthetic genes (113 KOs) suggests that the bacterial community may participate in nutrient cycles or provide precursors for the synthesis of plant compounds. Among these, we found entirely represented modules for the synthesis of tryptophan (M00023), lysine (M00016), proline (M00015) and methionine (M00017) in G-treated samples. Finally, the observed increase of both two-component system (TCS) (112 KOs) and bacterial secretion system (49 KOs) pathways, indicated that the community is highly responsive to environmental signals. These signals may originate not only from other bacteria competing to survive under newly established phyllosphere conditions but also from the plant itself following G peptide application.

### TCS analysis highlights potential mechanisms of interaction with the plant host

Because the TCS plays a central role in bacterial environmental sensing and adaptation, we further analyzed genes associated with this pathway. Specifically, we filtered the eggNOG gene table for TCS-associated KEGG terms to conduct a beta diversity analysis (Additional file 1: Fig. S2A). Results showed a clear separation between G-treated and CTRL plants (*R*^*2*^ = 0.300; *P* = 0.001). Differential abundance analysis (log_2_FC > 2 & < −1; *FDR* < 0.05; edgeR) revealed a significant enrichment (*n* = 175) rather than depletion (*n* = 10) following G treatments within the TCS-associated genes. Specifically, we identified several genes involved in secretion and extracellular structure formation (Additional file 1: Fig. S2B; Additional file 3: Table S6). Among them, *wza* (K01991), a polysaccharide biosynthesis export protein, and *TolC* (K12340), a component of the type I secretion system. In addition, the endoglucanase gene *egl* (K01179), which is involved in the degradation of plant polysaccharides, was highly abundant (log_2_FC = 5.45), suggesting a role in leaf colonization or nutrient recycling. The data also highlighted enriched gene sets associated with chemotaxis (mcp, cheA, cheW, cheR, cheB: K03406, K03407, K03408, K00575, K13924), quorum sensing (qseB, qseC: K07666, K07645), and biofilm formation (envZ, ompR, ompF: K07638, K07659, K09476), respectively. Moreover, genes involved in phosphate assimilation (phoA, K01077) and uptake (phoB, K07657; phoR, K07636), were also observed in G-treated plants. Most enriched genes were predominantly affiliated with Alphaproteobacteria and Gammaproteobacteria, with Sphingomonadaceae (42%) and Moraxellaceae (28%) being the most represented families.

### “Sphingobium yanoikuyae”: beyond bioremediation

To characterize bacterial taxa potentially driving the observed community shifts, we reconstructed metagenome-assembled genomes (MAGs) and profiled their plant growth-promoting traits (PGPTs). MAG identifiers (MAG ID) reflect the treatment in which each genome was predominantly detected (e.g., G for peptide-treated samples). Among 47 MAGs with completeness over 50% and contamination below 10% (Additional file 3: Table S7), dereplication resulted in 20 representative MAGs. Their abundance across samples and treatments was estimated as RPKM values (Additional file 3: Table S8; Additional file 1: Fig. S3). *Sphingobium yanoikuyae* (MAG ID: G_10) and *Acinetobacter johnsonii* (G_1) were among the most enriched MAGs in the G peptide-treated samples (Additional file 1: Fig. S3).

**Fig. 7:**
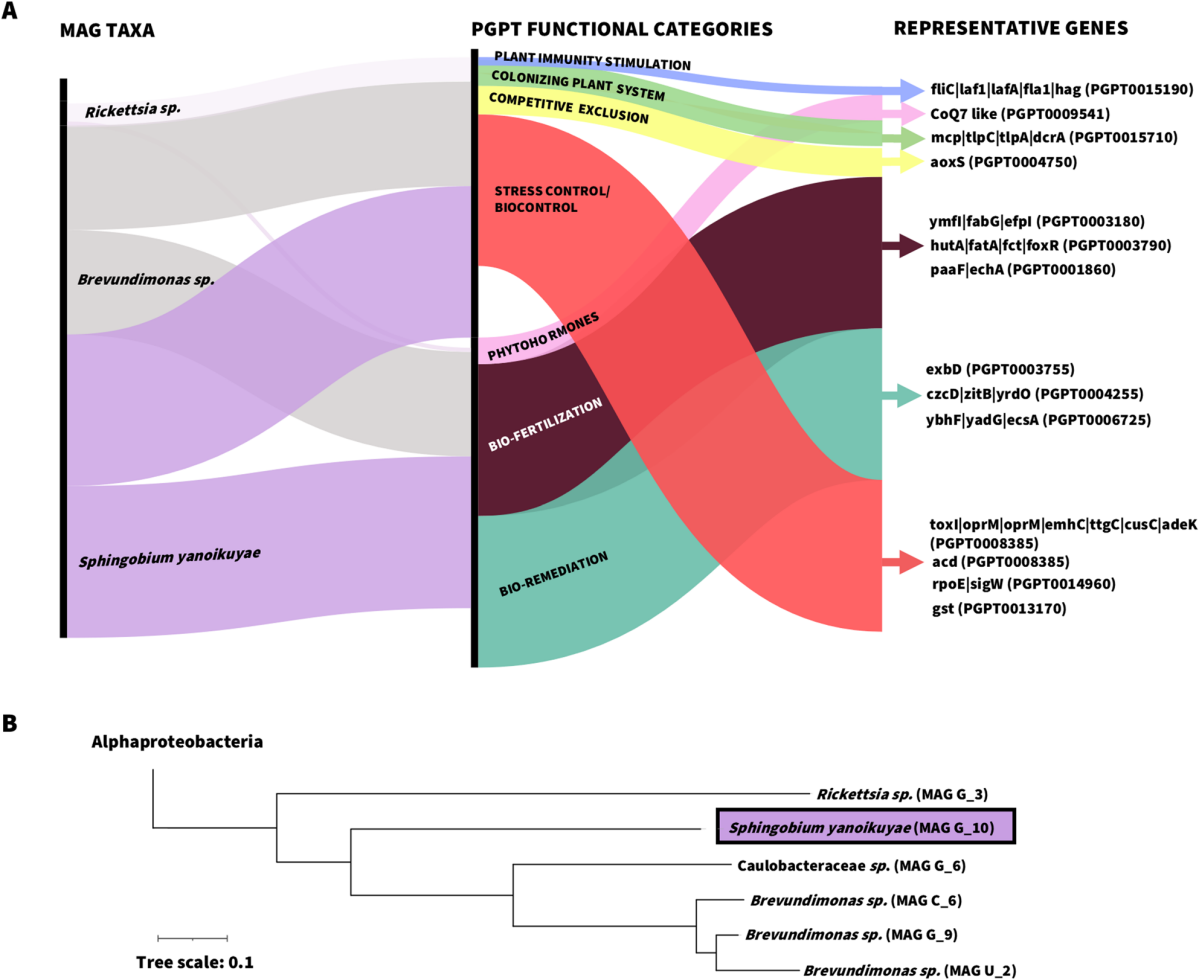
**Peptide-associated MAGs and their plant beneficial traits (PGPTs) according to PLaBAse.** Alluvial diagram (**A**) linking MAG taxa enriched in peptide-treated plants to plant growth-promoting trait (PGPT) functional categories and representative marker genes. PGPT categories correspond to level 2 of the PLaBAse PGPT-Pred functional assignment (plant immunity stimulation, colonizing plant system, competitive exclusion, phytohormones, bio-fertilization, bioremediation, and stress control/biocontrol), while representative genes correspond to level 6 annotations. Colors represent PGPT functional categories, and the thickness of the connections between levels is proportional to the number of genes assigned to each category. Phylogenetic placement of Alphaproteobacteria MAGs (**B**) reconstructed from metagenomic assemblies. The MAG corresponding to *Sphingobium yanoikuyae* (MAG G_10) is highlighted.

We next assessed PGPTs within the microbial communities promoted by the peptide using the PGPT-Pred module from the PLaBAse resource [47], focusing on high-quality dereplicated MAGs (completeness > 90%, contamination < 5%). The PGPTs-associated genes identified in each MAG and their functional assignments (from level 1 to 6) [47] are reported in Additional file 3: Table S9. *Sphingobium yanoikuyae* (MAG ID: G_10) harbored the highest number of predicted PGPT genes (1,398), followed by *Brevundimonas* (G_9) with 1,136 genes and *Rickettsia* (MAG G_3) with 443 genes (Fig. 7A, B). The phylogenetic relationships among reconstructed Alphaproteobacteria MAGs are shown in Fig. 7B, where *Sphingobium yanoikuyae* is most closely related to *Rickettsia* MAG.

*S. yanoikuyae* is commonly associated with bioremediation capabilities, including detoxification of heavy metals and xenobiotics. Accordingly, the PGPT-Pred tool assigned 115 genes to “heavy metal detoxification” and 73 to “xenobiotics biodegradation” to this MAG, compared to *Brevundimonas* (91 and 35) or *Rickettsia* (30 and 13), respectively (Additional file 3: Table S9). As shown in Fig. 7A, S. *yanoikuyae* and *Brevundimonas* displayed similar PGPTs functional assignments, with genes mainly involved in plant colonization (26%), stress-related biocontrol (20% and 21%, respectively), and plant nutrition (14%) (Additional file 3: Table S9).

Notably, *S. yanoikuyae* showed the highest gene count (*n* = 75) for hutA, fatA, fct, and foxR (PGPT0003790), genes encoding TonB-dependent receptors involved in iron uptake (Additional file 3: Table S9). These features suggest potential roles in biotic and/or abiotic stress resilience beyond its previously recognized bioremediation activities [48–50].

### Peptide triggers defense-associated reprogramming of tomato volatile metabolism

**Fig. 8:**
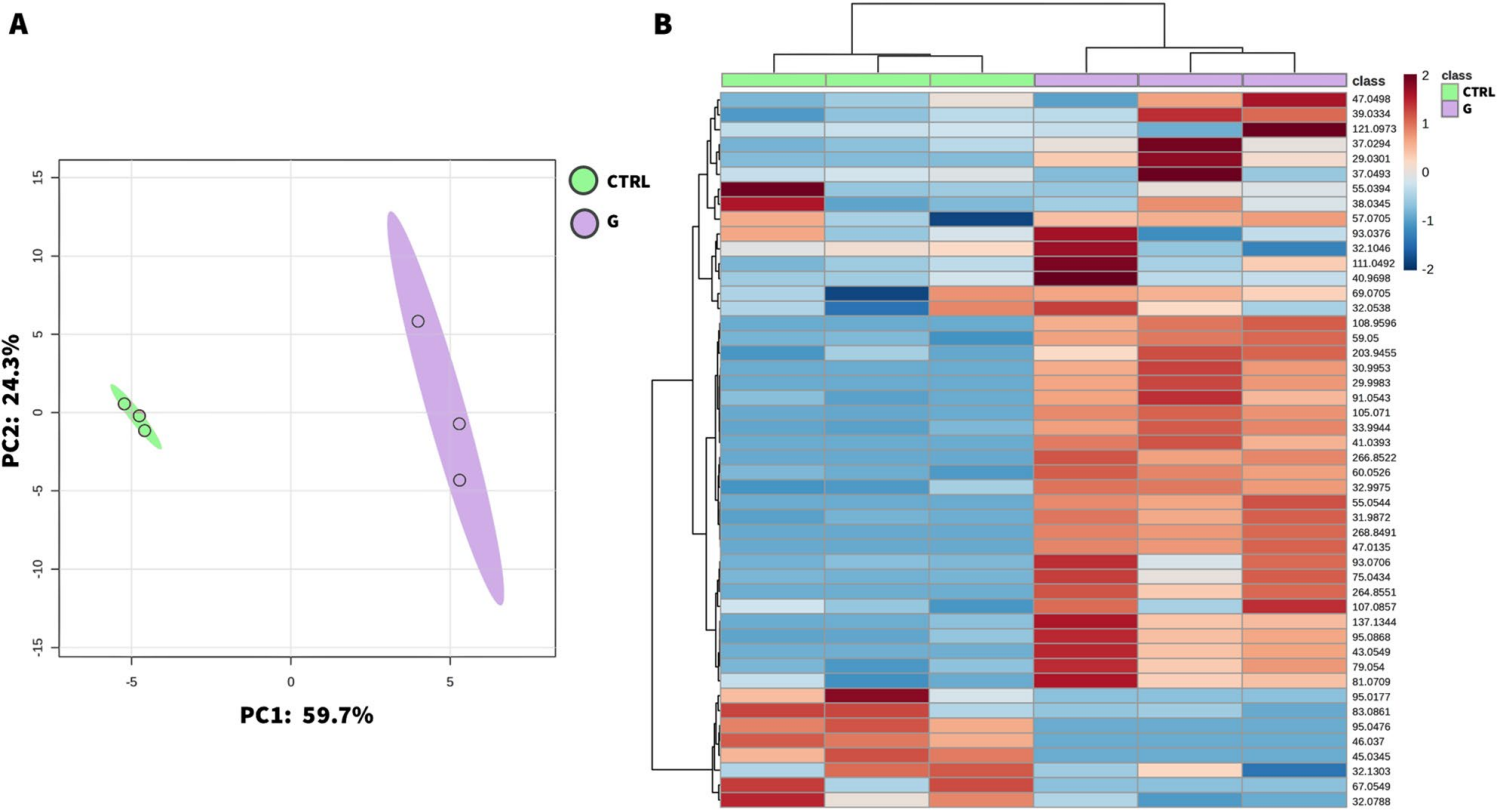
**Peptide G treatment distinctly reprograms tomato volatile organic compounds (VOCs) emissions.** Partial least squares discriminant analysis (PLS-DA) of VOCs emitted by tomato leaves 24 h after treatment with peptide G (purple) or control PBS 0.1X (green) (**A**). Hierarchical clustering heatmap based on Pearson distance and Ward’s linkage) of all detected VOCs (**B**). Columns represent biological replicates, and rows correspond to individual VOCs. Each colored cell indicates a normalized emission value according to a blue–red chromatic scale (from − 2, low emission, to + 2, high emission)

Previous studies have shown that the Prosystemin-derived peptide systemin can prime plant defenses at multiple levels, including the emission of VOCs, key mediators of plant ecological interactions [39, 51]. To explore whether the G peptide similarly affected the tomato volatilome, we profiled the leaf-emitted VOCs 24 h after peptide or CTRL treatments, as performed for metagenomic sampling time. A total of 48 VOCs were detected within the protonated mass range *m/z* = 20–300 after background subtraction of ion-source peaks and isotopes. The detected ions, their putative compound identification based on the PTR library, and corresponding molecular formulas are listed in Additional file 3: Table S10. The PLS-DA analysis clearly separated G-induced VOC profiles from the control, with the first two principal components explaining 84% of the total variance (Fig. 8A).

Hierarchical clustering further highlighted distinct VOC emission profiles between treatments (Fig. 8B; Additional file 3: Table S10). Several compounds showed increased abundance in G-treated plants, including monoterpenes such as α-pinene/β-ocimene/limonene/linalool (137.1344 m/z) and their fragments (95.0868 m/z; 81.0709 m/z). Ethylbenzene (107.0857 m/z), toluene (93.0706 m/z), and styrene (105.071 m/z) were also enriched. These VOCs are typically associated with defense responses, particularly within JA-related defense metabolism and Prosystemin signaling [51, 52]. In contrast, only a few low-molecular-weight compounds, mostly aliphatic fragments such as cyclohexene (83.0861 m/z), phenol (95.0476 m/z), and 2-pentenal (67.0549 m/z), were more abundant in CTRL samples. A t-test (*P* < 0.05) identified 16 ions with significantly different abundances between treatments, including monoterpenes and their fragments. The statistical values, compound identification, and enrichment patterns across treatments are reported in Additional file 3: Table S11. Together, these results indicate that G treatment alters the leaf volatilome, potentially modifying the chemical environment of the phyllosphere.

## Discussion

Treatment with the bioactive 16-amino acid G peptide [13] shifted the epiphytic phyllosphere microbiome of tomato plants, as indicated by a clear separation in beta diversity among experimental groups and reduced alpha diversity. Although higher microbial diversity is often associated with a “protective” microbiome, this relationship remains challenging to predict across plant species and environmental contexts [53, 54]. For instance, γ-aminobutyric acid (GABA), which enhances plant resistance and stimulates VOCs emission, reduced microbial diversity while promoting a suppressive activity against *southern leaf blight* [55]. Similarly, repeated foliar application of small peptides decreased bacterial richness in the tea phyllosphere while favoring taxa associated with plant growth promotion and defense responses [35]. Together, these observations suggest that diversity alone is not a reliable indicator of microbiome functionality. Instead, community composition and functional traits may better reflect microbiome contributions to host performance [53]. In our study, the ProSys-derived G peptide was associated with enrichment of Alphaproteobacteria, including *Sphingomonas*, *Sphingobium*, and *Brevundimonas*, as well as Gammaproteobacteria such as *Acinetobacter*, and Betaproteobacteria including *Delftia* and *Massilia*. These genera have been reported to support plant growth and stress tolerance [56, 57]. We also observed increased abundance of taxa commonly recognized as insect endosymbionts (e.g., *Portiera*, *Wolbachia*, *Rickettsia*, *Hamiltonella*), which are recurrently detected in plant tissues [58–60]. Some species, such as *Rickettsia belli*, can modulate salicylic acid (SA) and JA signaling in plants, contributing to viral and fungal pathogen suppression [61]. However, their ecological role within the plant holobiont remains unclear [62].

Network analysis further revealed that Proteobacteria and Actinomycetes were major contributors to bacterial connectivity in the tomato phyllosphere. In the CTRL network, these groups formed smaller, modular and relatively isolated subnetworks, suggesting more compartmentalized ecological niches. In contrast, peptide-treated samples displayed higher connectivity and a greater proportion of negative correlations, with Alphaproteobacteria and Betaproteobacteria emerging as network hubs. Increased number of negative correlations is consistent with niche differentiation or competitive interactions within structured communities [63, 64]. In addition, Proteobacteria are known to respond more rapidly to environmental changes than Actinobacteria [46, 65]. Under host-driven pressures, such as immune activation, tolerant or early-colonizing taxa may play a stronger role in shaping microbiome assembly, leading to shifts in hub identity and network cohesion [45]. Although co-occurrence networks do not demonstrate causality, the observed patterns may reflect increased resilience of the host-associated community to environmental perturbations [66].

Plant metabolic activity is known to influence microbial community composition, as changes in secondary metabolites can favor taxa adapted to the modified chemical environment while disadvantaging others [67, 68]. Accordingly, exogenous treatments can alter local plant microhabitats and host chemistry [53, 69], thereby influencing microbiome composition. For instance, application of the signaling peptide RALF23 enriched fluorescent pseudomonads in the *Arabidopsis* rhizoplane while suppressing *Fusarium oxysporum* [38]. In our system, it remains to be determined whether peptide-shaped interactions similarly contribute to the suppression of foliar pathogens. Nevertheless, our observations are consistent with reports in other plant species showing that activation of JA-mediated defenses can modulate microbiome dynamics. For example, JA-dependent responses in wheat [70] or rice [71] are associated with reduced bacterial diversity in root microbiomes, whereas JA-deficient *Arabidopsis* mutants exhibit increased diversity [72]. Likewise, exogenous JA treatments decrease Shannon evenness in the bittercress phyllosphere [73], supporting the idea that defense-related molecules, whether endogenous or externally applied, can reshape plant-associated microbial communities. ProSys and its derivatives activate JA-related pathways and induce the production of secondary metabolites including phenylpropanoids [14, 40, 74, 75], which are known to influence microbiome assembly [76]. In addition to soluble metabolites and exudates, VOCs represent another major class of signaling molecules that can influence ecological interactions by attracting beneficial organisms or deterring threats [39]. Consistent with defense activation, our volatilome analysis revealed increased emission of monoterpenes and their derivatives (e.g. α-pinene, β-myrcene, limonene, and β-ocimene) in G-treated plants. These profiles resemble those observed in ProSys-overexpressing, JA-treated, or herbivore-stressed tomato plants [51, 52, 77]. Such volatile blend can be detected as early as 24 h after JA induction [40, 51, 78], following the upregulation of *LOX* and *AOS* genes [13, 14, 16] by ProSys-derived peptides. Within plant–microbe interactions [60, 77], VOCs can be metabolized or transformed by host-associated microbes [79], suggesting that blend emissions can represent one of several factors influencing microbial composition. In this context, members of the Sphingomonadaceae family may be metabolically suited to persist under conditions characterized by higher terpenoid production. Indeed, this group is frequently associated with leaf glandular trichomes [44], recognized sites of terpenoid release. Moreover, *S. yanoikuyae* harbors gene clusters involved in limonene and α-pinene detoxification [80, 81] detected upon G peptide treatment. A direct causal link between G-induced VOCs emissions and the observed microbiome shifts cannot be established from the present data; nonetheless, the concordance between the detected terpenoids and the metabolic potential of Sphingomonadaceae is intriguing and warrants targeted mechanistic testing.

In parallel, other JA-associated secondary metabolites could also contribute to Sphingomonadaceae persistence in the leaf environment. They can degrade α-tomatine [82–84] that is associated with JA induction [85, 86]. Tomato lines with impaired JA signaling show reduced tomatine levels, lower Sphingomonadaceae abundance, and increased susceptibility to herbivory [87]. Tomatine has been linked not only to the recruitment of Sphingomonadaceae, but also to decreased abundance of some Actinobacteria and reduced Shannon diversity [82–84]. Although tomatine was not quantified here, these observations raise the possibility that JA-associated secondary metabolites contributed to Sphingomonadaceae persistence under peptide treatment. Consistent with a JA-mediated shifts in Actinomycetes, Methyl jasmonate (MeJA) treatment in wheat or tomato decreased *Streptomyces* [70, 88], that instead increased in *Arabidopsis* JA-deficient mutants [89]. Scopoletin, a MeJA-induced phytoalexin [90], inhibited the growth of the pathogen *Streptomyces scabiei* [76], a close relative of *S. caniscabiei* [91], whose abundance was significantly reduced by peptide treatment in our study (Additional file 3: Table S2). The observed reduction in Actinomycetes, particularly *Streptomyces*, should be interpreted with caution, as these taxa are widely recognized for roles in plant growth promotion and tolerance to stress [70, 92]. In addition, the single time-point sampling design of this study limits our ability to capture the temporal dynamics and the persistence of these shifts. Plant developmental stage, seasonality, soil properties, and microclimatic conditions, could indeed act as confounding variables [93–96]. The 24 h sampling time was selected to capture downstream metabolic responses in the leaf environment [40, 52, 78] that are more likely to influence microbiome assembly than early transcriptional responses [97, 98]. In addition, repeated foliar applications maintain peptide treatment effects over time [41], which can in turn shape microbiome dynamics [35].

Functional profiling associated with G application revealed enrichment of bacterial motility and biofilm formation pathways, resembling patterns after MeJA treatment in tomato plants [60]. Among the most abundant genes, *wza* (K01991) is involved exopolysaccharides (EPSs) extrusion, which is crucial for biofilm formation [99, 100]. Within the TCS signaling pathway (K02020), both EPSs and quorum sensing are essential processes for plant surface colonization and protection against salinity, drought, and pathogen attack [101–103]. TCS signaling plays a central role in bacterial responses to environmental stress [68, 104]. Together with bacterial secretion system, it has been linked to the beneficial roles of *Sphingomonas* and *Bacillus*, which can be recruited by cucumber root exudates during *Fusarium oxysporum* infection [104]. KEGG-KO enrichment analysis also showed how the peptide increased pathways related to the biosynthesis of cofactors, vitamins, and amino acids. Similar functional enrichments were described in the rhizosphere microbiome of cowpea under herbivory stress by the leafminer *Liriomyza trifolii* [105]. Vitamins of the B group and amino acid biosynthesis by plant-associated microbes contribute to plant growth, defense, and adaptive responses [23, 106–109]. For example, enhanced tryptophan biosynthesis is associated with JA-mediated responses against *Spodoptera littoralis* [110], whereas its levels decrease in the root exudates of plants with impaired JA production [89]. Among genes enriched under G peptide treatment, we also detected *nif* and *fix* genes involved in N fixation, as well as *nir* genes associated to N assimilation, both linked to *Acinetobacter* and *Sphingobium* activities [105, 111, 112]. These observations support the hypothesis that plants may adopt a “cry-for-help” strategy, reallocating nitrogen to support the synthesis of defense related compounds [105].

Our findings suggest that members of Sphingomonadaceae, particularly *S. yanoikuyae*, may be favored under peptide application, potentially due to their metabolic versatility. Moreover, their reported activity against foliar pathogens [104] is consistent with the enrichment of TonB-dependent receptor genes, which have been linked to pathogen suppression in *Sphingomonas* strains [103].

## Conclusion

Our findings demonstrate that application of the ProSys-derived peptide induced a significant and targeted reshaping of the tomato phyllosphere microbiome, affecting both its taxonomic composition and functional potential. This remodeling may be driven by a change in the defense leaf chemicals as shown by distinct VOCs emission following peptide application. Consistent with the known effects of Prosystemin on fungal pathogens and insect herbivores, the observed microbiome shifts resemble those reported in plants with activated JA defenses. Because host-associated microbiomes are highly dynamic, longitudinal analyses will be required to determine whether these changes are transient or persist over time. Integrating temporal sampling with environmental and host-related defense response will help clarify the ecological significance of these interactions. Ultimately, these results motivate further work on peptide-based strategies for sustainable crop protection, including microbiome-informed experiments.

## Methods

### Plant material and growth conditions

In April 2023, *Solanum lycopersicum L.* cultivar “*San Marzano nano*” tomato seeds were germinated in a growth chamber at a controlled temperature of 24 ± 1 °C, 60 ± 5% relative humidity (RH) and complete darkness. The seeds were placed in standard 90 mm Petri dishes (Thermo Fisher Scientific, USA), with approximately 20 seeds per dish on moist, sterile paper for a few days until the emergence of the rootlets. The plantlets were subsequently moved to a polystyrene tray containing autoclaved soil mixed in a greenhouse growth chamber at 26 ± 1 °C with 60 ± 5% RH and an 18:6 h light/dark photoperiod. Following an adaptation period of two weeks, the plants were transplanted into 9-cm-diameter pots filled with autoclaved soil mixture and kept under the same conditions until they reached two months of growth.

### Treatment experimental design

For metagenomic analyses, two-week-old plants were sprayed every 15 days, for a total of four treatments, with the spray volume adjusted according to plant growth and size. The application schedule was inspired by the frequency and timing previously adopted under field conditions [41]. The experimental plan included the three following treatments: 100 fM ProSys-derived peptide (referred to as G) diluted in 0.1X phosphate-buffered saline (PBS) as previously reported [13], 0.1X simple PBS representing the control (CTRL) and untreated plants (UNT). The synthetic peptide used was obtained as previously described [13]. The total amount of spray across all the treatments combined was 13 mL per plant, achieving comprehensive coverage of each plant aerial part at every exogenous application. Among a total of 75 tomato plants, 30 were used for each of the G and CTRL experimental groups, and 15 were used for the UNT group. Each biological replicate consisted of 12 compound leaves collected from three independent plants (4 leaves per plant) belonging to the same treatment group. Fully expanded and healthy leaves were selected from mid-canopy positions across the three plants, avoiding both the youngest apical and the oldest basal leaves to ensure comparable developmental stages. For the downstream analyses, leaf samples were collected once at a single time point, 24 h after different experimental treatments.

For volatilome profiling, six plants were grown and treated under the same experimental conditions, with three plants receiving 100 fM G peptide and three treated with 0.1× PBS (control). Unlike the metagenomic experiment, these plants received a single foliar application, and volatile collection was performed 24 h post-treatment, corresponding to the same time point used for metagenomic sampling.

### Leaf sample processing and microbial DNA extraction

Leaf microbial communities were collected following Gupta et al. [20], with minor modifications. From each selected compound leaf, all five leaflets were excised using ethanol-sterilized scissors, corresponding to approximately 20 leaflets per plant and 60 leaflets per biological replicate (three pooled plants). The excised leaflets were placed in sterile Ziplock bags and kept on ice until further laboratory processing. To isolate epiphytic microbes, 240 mL of 0.1 M sterilized PPB (Potassium Phosphate Buffer containing K₂HPO₄ and KH₂PO₄ in equimolar proportion; pH 8) was added, followed by gentle manual shaking, 5 min sonication (50 kHz, Falc Instruments, Treviglio, Italy), and 30 s vortexing. This process was repeated twice. The resulting washing mixture was centrifuged at 11,000 *× g* for 20 min at 4 °C, and collected microbial pellets were resuspended in PPB, transferred into 2 mL tubes, and centrifuged at 14,000 *× g* for 2 min at 4 °C. Pellets were stored at −20 °C until DNA extraction, which was performed using DNeasy PowerSoil Pro Kit (Qiagen, Hilden, Germany) according to the manufacturer’s instructions and quantified with the Qubit HS Assay (Thermo Fisher Scientific, Waltham, Massachusetts, United States).

### Quantitative Real-Time PCR (qPCR) analysis of bacterial abundance

Total bacterial abundance and selected taxa were quantified using SYBR Green-based qPCR with universal primers 515f–806r [113], and taxon-specific primers for Alphaproteobacteria (ALF28f/ALF986r), Gammaproteobacteria (Gamma395f/Gamma871r), Actinobacteria (243f-513r), and *Sphingomonas* (Sph-spt694f-Sph-spt983r) [114–117], amplifying a gene for serine palmitoyltransferase (*spt*). Reaction mixtures contained 1 µL of extracted DNA, 5 µL KAPA SYBR^®^ FAST qPCR Master Mix 2X (KAPA Biosystems, USA), 1 µL of each 10 µM primer, and 3 µL ultrapure water. Amplification was performed on a Q-Tower 3 (Analytik Jena, Germany), with an initial denaturation step at 95 °C for 10 min, followed by 40 cycles of denaturation at 95 °C for 30 s, primer-specific annealing for 30 s at 54 °C (total bacteria, Alpha-, and Gammaproteobacteria), 63 °C (Actinobacteria), or 55 °C (*Sphingomonas*), extension at 72 °C for 30 s, concluding with a final melting curve analysis. Differences in the abundance of bacteria, among the three different conditions (UNT, CTRL, G) were evaluated using Dunn’s test on log_10_ transformed bacterial gene copy number data.

### Shotgun metagenomic sequencing and analysis

Shotgun metagenomic sequencing was performed by the sequencing provider Novogene Co., Ltd. (Beijing, China). Libraries were prepared using the Nextera XT Index Kit v2 (Illumina, San Diego, California, United States), and sequencing was performed on an Illumina NovaSeq platform (Novogene Europe), leading to 2 × 150 bp reads. Of the 25 samples submitted for sequencing, 22 were successfully processed, resulting in 10 samples for the G treatment, 8 for the CTRL group, and 4 for the UNT group. Quality control of raw reads involved adaptor trimming and quality filtering (Phred < 20) using Trimmomatic v0.39 [118] and VSEARCH v2.15.2 [119]. Host DNA contamination was removed by aligning reads to the *Solanum lycopersicum* reference genome (GCF_000188115.5) using Bowtie 2 [120]. Unmapped reads were assembled with MEGAHIT v1.2.9 [121], retaining contigs > 1 kb.

Taxonomic classification and species abundance estimation were conducted using Kraken2 v2.0.9 and Bracken v2.6.0 [122, 123]. Open reading frames (ORFs) were predicted with Prodigal v2.6.3 [124], and a non-redundant, protein-coding gene catalog was built using CD-HIT-EST v4.8.1 [125] (nucleotide identity cutoff 95%). Gene function and taxonomy were assigned using DIAMOND and eggNOG-mapper [126, 127] against the eggNOG v5.0 database [128]. Gene abundances were estimated by mapping back quality-filtered reads to the non-redundant, protein-coding gene catalog using BWA v0.7.17 and SamTools v1.7 [129, 130].

### Binning of bacterial metagenome-assembled genomes (MAGs) and PGPB trait prediction

MAGs were reconstructed using Maxbin2 v2.2.7, MetaBAT2 v2.12.1 and CONCOCT v1.1.0 assembly tools [131–133]. Quality assessment was performed using CheckM v1.0.13 [134], retaining medium-quality bins (completeness > 50%, contamination < 10%), then dereplicated via both DASTool v1.1.1 [135] and DRep [136]. Taxonomic classification and functional annotation were conducted with GTDB-Tk [137] and DRAM [138]. Abundance profiles for each MAG were determined using CoverM v0.4.0 with -rpkm mode [139] while phylogenetic relationships among MAGs were analyzed using PhyloPhlAn database [140] to construct a phylogenetic tree. Protein FASTA sequences of dereplicated, high-quality MAGs (completeness > 90% and contamination < 5%) from selected bacterial species were further used for predicting plant growth-promoting traits with the PLaBAse PGPT-Pred tool [47] in strict mode (BLASTP + HMMR).

### Characterization of bacterial diversity and composition

Alpha diversity analysis was performed by normalizing the abundance data to the lowest number of reads to adjust for unequal sequencing depth. The abundance data were normalized using MetagenomeSeq’s cumulative sum scaling (CSS) [141] and subsequently used for beta diversity analysis. The microbial datasets were analyzed with the R packages Phyloseq, MicrobiomeAnalyst, and vegan, all implemented in RStudio [142–145]. Significant differences in alpha diversity metrics (observed species, Shannon index, and Pielou’s evenness) were assessed via the Kruskal‒Wallis test as a nonparametric approach. Differences in community composition between groups were investigated using a normalized Bray‒Curtis dissimilarity matrix and further evaluated by a Permutational multivariate analysis of variance (PERMANOVA) using adonis2 function implemented in the vegan package [144, 145]. Differential abundance of bacterial taxa or genes in metagenomes were explored using the edgeR package v4.2.0 [146, 147]. Results were considered significant at P_adjusted_ value < 0.05, filtered for log_2_-fold change (log_2_FC) > 2 for enrichment and log_2_FC <−1 for depletion, and with a relative abundance of bacterial species > 0.05%. Bacterial species defined as “core” were identified within each of treatment groups to capture the most stable and recurrent taxa characterizing each condition. The analysis was performed using the *core_members* function of the microbiome R package [148], setting a detection threshold of ≥ 0.1% for relative abundance and a prevalence threshold of ≥ 75% of samples within each group). Subsequently, shared core species were determined as the intersection among the condition-specific cores, visualized in the Venn diagram (Fig. 3B) and listed in Table S3.

### Co-occurrence bacterial network and functional analysis of pathways

Bacterial taxa were filtered to retain those present in at least five samples and with a total relative abundance ≥ 0.1%. Network inference was performed using the NetCoMi R package (v1.0.5) [149]. Sparse inverse covariance matrices were estimated with the SPRING algorithm (Stability-based Relative Inference of Networks) which infers conditional dependencies among taxa while accounting for compositional effects. The model was parameterized with *nlambda = 30*, *lambda.min.ratio = 0.3*, and *Rmethod = “approx”* to balance sparsity and stability. Also, the network selection was based on the highest frequency function (100), to select the most important nodes. Topological properties of the resulting networks were computed both NetCoMi and Gephi software [150]. Putative influential taxa (hubs) were defined as nodes exceeding the 95th percentile of the fitted log-normal distribution of eigenvector centrality values within the largest connected component (LCC). Network layouts were generated using a force-directed Fruchterman–Reingold algorithm, with node size and edge thickness proportional to eigenvector centrality and association weight, respectively.

Functional enrichment analysis of KEGG Orthology (KO) terms was conducted using Microbiome Profiler v1.10.0 package [151], considering significantly enriched (log_2_FC > 2, FDR < 0.05) or depleted (log_2_FC < −1, FDR < 0.05) terms between CTRL condition and G-treated samples. KO pathway analysis was performed using KEGGREST package v1.44.1 in RStudio [152].

### Volatile organic compounds (VOCs) collection and analysis

Six tomato plants treated as described above were used to collect VOCs under a controlled temperature, at 25 ± 1 °C. Headspace VOCs sampling was performed 1 h after closing the plants in a glass box (60 × 60 × 60 cm) to accumulate VOCs. The collected headspaces were directly injected into the PTR-Qi-TOF-MS (Proton Transfer Reaction -Quadrupole interface- Time of Flight - Mass Spectrometer) drift tube hated (110 °C) peek inlet tube with a flow rate of 100 sccm. VOCs were detected in real-time through proton transfer reactions using Proton Transfer Reaction-Quadrupole interface Time of Flight- Mass Spectrometry (PTR-Qi-TOF-MS) apparatus supplied by Ionicon Analytik GmbH (Innsbruck, Austria). The drift tube was kept under controlled conditions of pressure (3.8 mbar), temperature (80 °C) and voltage (1000 V), resulting in a field density ratio (E/N) of 141 Td (E being the electric field strength and N the gas number density; 1Td = 10-^17^ V cm^2^). The VOCs protonation was carried out by using H_3_O^+^ as proton donor in the transfer reaction and was effective for VOCs having a proton affinity higher than that of H2O (691.7 kJ mol-1). The raw PTR-QiTOF-MS data were processed using the R package ptairMS [153]. Integrated peak signals from PTR-Qi-TOF-MS, in units of ppb, were used for further analysis. The m/z signals were blank corrected by subtracting the signal obtained from the empty glass bottle and the peaks associated with the PTR-MS ion source, including those ascribed to water chemistry or other interfering ions, for example, m/z = 32 (O2+), m/z = 31.022 (NO+), m/z = 37, and m/z = 39.033 (corresponding to H318O + and water cluster ions H2O-H318O+, respectively), were manually removed. Statistical analyses, including partial least squares-discriminant analysis (PLS-DA), hierarchical clustering, and univariate analysis (t-test), were performed using Metaboanalyst online platform [154] and R internal statistical functions. Statistics was performed on the VOCs data expressed in ncps (normalized counts per second), following normalization by log₁₀ transformation and autoscaling. Tentative identification (t.i.) of each mass peak detected by PTR-Qi-TOF-MS, was based on PTR libraries [155, 156].

## Supplementary Information

Supplementary Material 1. Figure S1: Amino acid sequences of NCs peptides and their effects on defense gene expression and *Spodoptera littoralis* larval performance. Figure S2. Two-component system overview of the tomato phyllosphere microbiome. Figure S3. Phylogenetic tree and abundance profiles of metagenome-assembled genomes (MAGs) across different treatment of tomato leaves.

Supplementary Material 2. Supplementary Methods: including gene expression analysis, primer sequences used for qPCR, and *Spodoptera littoralis* feeding bioassay performed for NCs peptides.

Supplementary Material 3. Table S1: Number of sequencing reads per sample. Table S2: Differential abundance analysis between peptide (G) and PBS0.1X-treated (CTRL) tomato phyllosphere samples. Table S3: Shared core and unique core bacterial species. Table S4: KEGG_KO enrichment analysis of bacterial genes associated with G peptide treatment. Table S5: Topological properties of bacterial networks. Table S6: differentially enriched bacterial genes associated to two-component system (TCS) pathway in G-treated samples compared to CTRL condition. Table S7: CheckM results with medium-quality assembled genomes (MAGs) and relative taxonomy classification. Table S8: Number of mapped Reads Per Kilobase per Million reads (RKPM) for each Metagenome-Assembled Genome (MAG) across the different treated samples. Table S9: PLaBAse annotation. Table S10: Putative identification of Volatile Organic Compounds (VOCs) collected from tomato plants leaves. Table S11: List of significant VOCs among G-treated and CTRL tomato plants.

## Data Availability

Phyllosphere metagenomes are available in the European Nucleotide Archive (ENA) ([http://www.ebi.ac.uk/ena]) under the project number PRJEB86145. Shotgun metagenome reads were deposited under accession numbers ERS23746230-ERS23746251. All the data generated or analyzed during this study are included in this published article and its supplementary information files.

## Abbreviations

ProSys: Prosystemin
UNT: Untreated leaves
CTRL: PBS 0.1X treated leaves
G: Prosystemin-derived peptide
KO: KEGG Orthology
VOCs: Volatile Organic Compounds
