# Supplementary material for: Prosystemin-derived signals: bridging leaf microbiome dynamics and defense activation
Source: Environ Microbiome. 2026 Apr 5;21:71. doi: 10.1186/s40793-026-00885-9 (PMC13188736; doi:10.1186/s40793-026-00885-9)
Supplement: Supplementary file 1 — Supplementary Material 1. Figure S1: Amino acid sequences of NCs peptides and their effects on defense gene expression and Spodoptera littoralis larval performance. Figure S2. Two-component system overview of the tomato phyllosphere microbiome. Figure S3. Phylogenetic tree and abundance profiles of metagenome-assembled genomes (MAGs) across different treatment of tomato leaves. [file 40793_2026_885_MOESM1_ESM.pdf]

Additional file 1: Supplementary figures

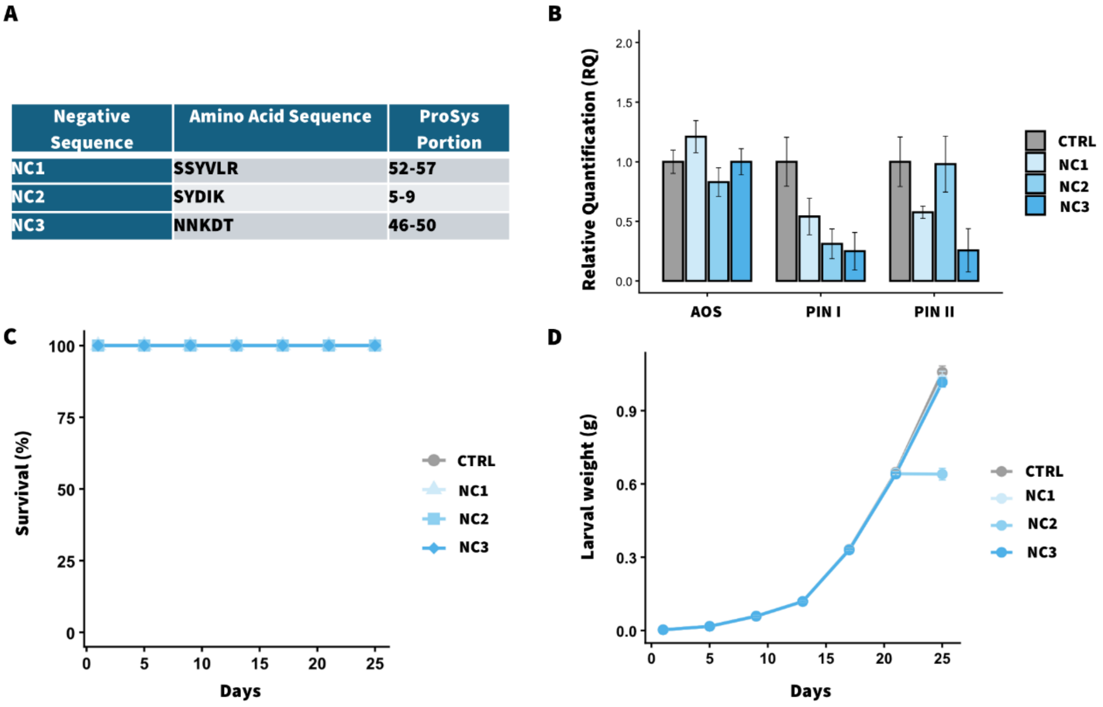

Figure S1. Negative control peptides derived from Prosystemin do not trigger defense responses in tomato. Amino acid sequences and positions of the three negative control peptides (NC1–NC3) (**A**) selected from regions of the ProSys protein lacking predicted functional motifs. Expression analysis of jasmonate-responsive defense genes (*AOS*, *PIN I*, *PIN II*) in tomato leaves (**B**) treated with 100 fM solutions of NC1, NC2, and NC3. Transcript levels were quantified by qRT-PCR 6 hours after treatment, normalized to the reference gene Ef-1α, and expressed relative to mock-treated plants (CTRL), set as 1. Letters indicate different statistical groups (One-Way ANOVA, $P < 0.05$). Error bars indicate ± standard error. Survival rate of *Spodoptera littoralis* larvae (**C**) fed with tomato leaves treated with NCs peptides (100 fM) over a 25-day period. Larval weight of *S. littoralis* (**D**) feeding on NCs-treated tomato leaves measured throughout the feeding assay. Third-instar larvae ($n = 32$ per treatment) were fed with treated leaf disks collected 6 h after peptide application. No significant differences between the NCs treatments and the mock (CTRL) were detected in both larval weight and survival rate (One-Way ANOVA, $P > 0.05$). Error bars represent ± standard error.

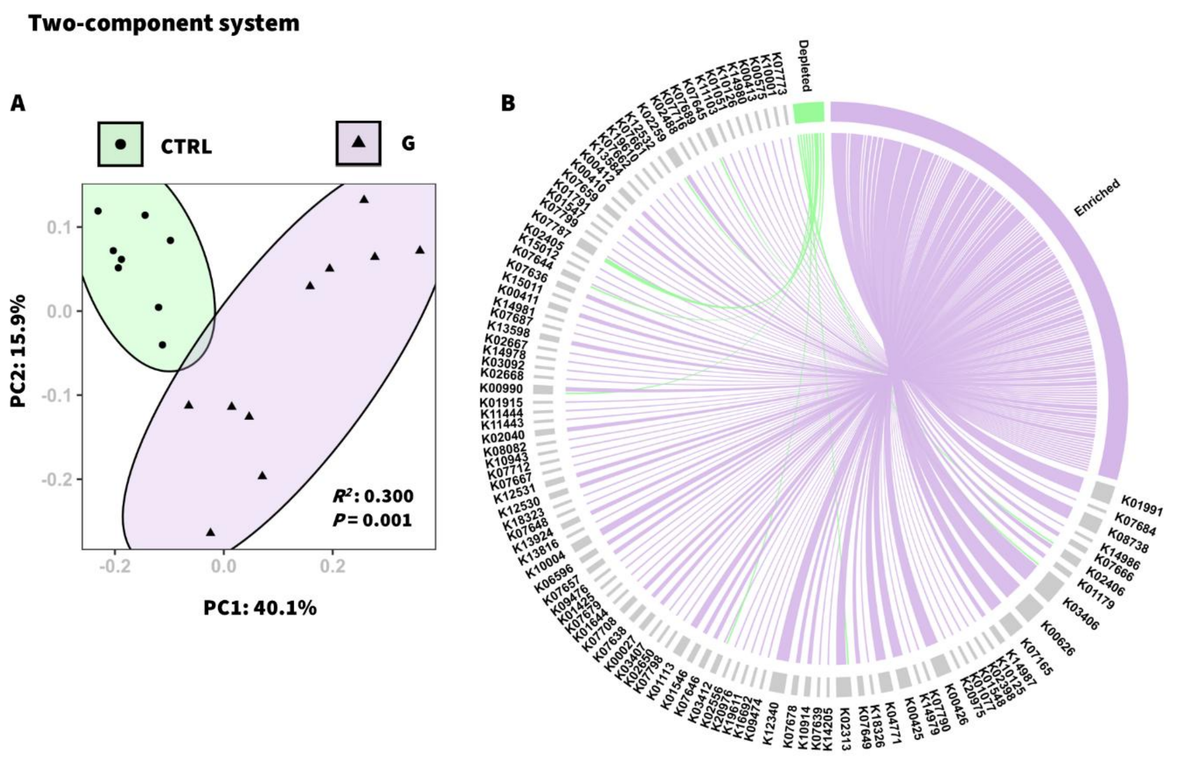

Figure S2. Two-component system overview of the tomato phyllosphere microbiome. PCoA based on Bray–Curtis metrics (**A**) showing differences between CTRL and G-treated samples based on the TCS-associated genes. Chord diagram (**B**) showing the connections between the source represented by genes classified as enriched or depleted in the peptide-treated samples ($log_2FC > 2$ or < -1; *FDR* < 0.05; edgeR) and the targets represented by associated KEGG terms related to the TCS pathway (B). Enriched or depleted KEGG terms are depicted in violet or green, respectively. The thickness of the chords represents the log_2FC values, which range from -1 to 7.92.

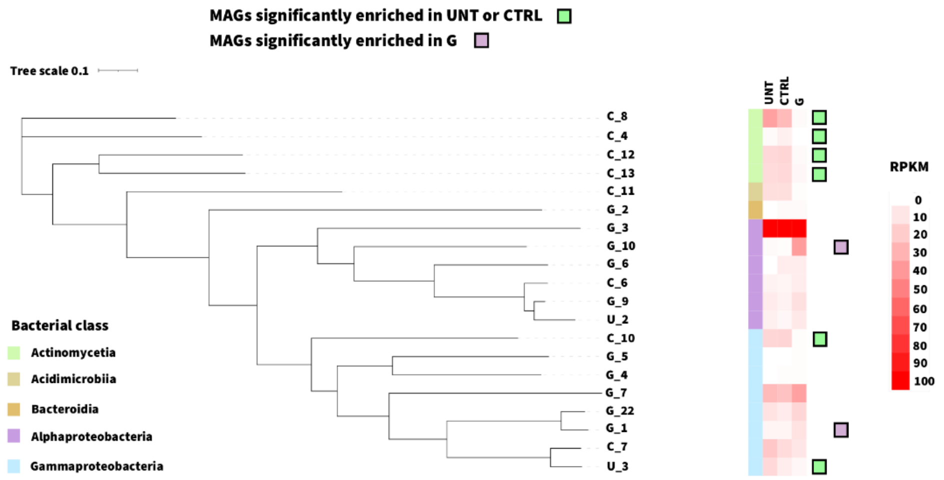

Figure S3. Phylogenetic tree and abundance profiles of metagenome-assembled genomes (MAGs) across different treatment of tomato leaves. Phylogenetic tree of dereplicated MAGs (completeness >50% and contamination <10%), colored according to bacterial class taxonomy. Abundance of Reads Per Kilobase per Million mapped reads (RPKM) values for each MAG across untreated (UNT), control (CTRL), or peptide-treated (G) samples, are reported in the right panel. Significantly enriched MAGs are represented by green squares for control conditions (UNT/CTRL) or violet squares for G peptide treatment.
