# Supplementary material for: Prosystemin-derived signals: bridging leaf microbiome dynamics and defense activation
Source: Environ Microbiome. 2026 Apr 5;21:71. doi: 10.1186/s40793-026-00885-9 (PMC13188736; doi:10.1186/s40793-026-00885-9)
Supplement: Supplementary file 2 — Supplementary Material 2. Supplementary Methods: including gene expression analysis, primer sequences used for qPCR, and Spodoptera littoralis feeding bioassay performed for NCs peptides. [file 40793_2026_885_MOESM2_ESM.pdf]

Additional file 2: Supplementary Methods

Plant treatments and gene expression analyses of NCs

Four-week-old tomato plants were treated with 100 NCs1-3 in 0.1X PBS. Specifically, the abaxial surface of fully expanded leaves was treated with 15 spots of 2μL od NCs or mock (0.1X PBS). Leaves from three biological replicates were collected 6 hours post treatment (hpt) and used for the downstream analyses. Total RNA extraction, synthesis of the first cDNA strand and qPCR steps were conducted as previously described [1]. Gene expression was normalized to the reference gene EF-1α [2], and relative transcript levels were calculated using the 2^{-}ΔΔCt method [3] following qPCR analysis on a Rotor-Gene 6000 system (Corbett Research; Sydney, Australia). Primers used for each gene are reported in Table SM1.

Table SM1: List of primers used for qPCR analysis for NCs gene expression.

Primer	Sequence (5'-3')	Gene symbol	Accession number
EF-1α Fw	CTCCATTGGGTCGTTTTGCT	*EF-1α*	Solyc06g005060
EF-1α Rv	GGTCACCTTGGCACCAGTTG		
AOS Fw	GATCGGTTCGTCGGAGAAGAA	*AOS*	Solyc11g069800
AOS Rv	GCGCACTGTTTATTCCCCACT		
PIN I Fw	GAAACTCTCATGGCACGAAAAG	*PIN I*	Solyc09g084470
PIN I Rv	CACCAATAAGTTCTGGCCACAT		
PIN II Fw	CCAAAAAGGCCAAATGCTTG	*PIN II*	Solyc03g020060
PIN II Rv	GTGCAACACGTGGTACATCCT		

Insect feeding bioassay

Spodoptera littoralis (Lepidoptera, Noctuidae) larvae were reared on an artificial diet until they reached the third instar stage, as previously described [4]. The diet consisted of 41.4 g L^{-1} wheat germ, 59.2 g L^{-1} brewer's yeast, 165 g L^{-1} corn meal, 5.9 g L^{-1} ascorbic acid, 1.53 g L^{-1} benzoic acid, 1.8 g L^{-1} methyl-4-hydroxybenzoate, and 29.6 g L^{-1} agar. Larvae were maintained under controlled conditions at 25 ± 1 °C, 70 ± 5% relative humidity, and a 16:8 h light/dark photoperiod. Tomato leaves were collected 6 hpt with NCs and cut into leaf disks. Larvae were placed in 4-well plastic rearing trays (RT32W, Frontier Agricultural Sciences, Pitman, NJ, USA) containing 3 mL of 1.5% (w/v) agar-agar to maintain leaf turgidity. Leaf disks were placed in each well and adjusted daily in sizes according to the feeding requirements of the developing larvae. For each treatment, 32 larvae were monitored. Larval weight and mortality were recorded daily until pupation.

Statistical analyses

Gene expression was analysed using two-way ANOVA procedure with Tukey's or Duncan post-hoc test ($P<0.05$). Survival curves of *S. littoralis* were analysed by using Kaplan–Meier and Log-rank approach. Larval weights were evaluated using One-Way ANOVA test ($P<0.001$).

References

1. Castaldi V, Langella E, Buonanno M, Di Lelio I, Aprile AM, Molisso D, et al. Intrinsically disordered Prosystemin discloses biologically active repeat motifs. Plant Sci. 2024;340:111969. https://doi.org/10.1016/j.plantsci.2023.111969.

2. Marum L, Miguel A, Ricardo CP, Miguel C. Reference Gene Selection for Quantitative Real-time PCR Normalization in Quercus suber. PLOS ONE. 2012;7:e35113. https://doi.org/10.1371/journal.pone.0035113.

3. Livak KJ, Schmittgen TD. Analysis of relative gene expression data using real-time quantitative PCR and the 2-ΔΔCT method. Methods. 2001;25. https://doi.org/10.1006/meth.2001.1262.

4. Di Lelio I, Varricchio P, Di Prisco G, Marinelli A, Lasco V, Caccia S, et al. Functional analysis of an immune gene of Spodoptera littoralis by RNAi. J Insect Physiol. 2014;64:90–7. https://doi.org/10.1016/j.jinsphys.2014.03.008.
